# Probiotic Sonicates Selectively Induce Mucosal Immune Cells Apoptosis through Ceramide Generation via Neutral Sphingomyelinase

**DOI:** 10.1371/journal.pone.0016953

**Published:** 2011-03-09

**Authors:** Sandra Angulo, Albert Morales, Silvio Danese, Laura Llacuna, Maria Carme Masamunt, Nicole Pultz, Maria Grazia Cifone, Claudio De Simone, Salvadora Delgado, Jordi Vila, Julián Panés, Curtis Donskey, Jose C. Fernández-Checa, Claudio Fiocchi, Miquel Sans

**Affiliations:** 1 Department of Gastroenterology, Hospital Clinic, IDIBAPS, CIBER Enfermedades Hepáticas y Digestivas (CIBERehd), Barcelona, Spain; 2 Liver Unit, Hospital Clinic, IDIBAPS, CIBERehd, Barcelona, Spain; 3 Department of Cell Death and Proliferation, Instituto de Investigaciones Biomédicas de Barcelona (IIBB), Consejo Superior de Investigaciones Científicas (CSIC), Barcelona, Spain; 4 Division of Gastroenterology, Istituto Clinico Humanitas, Milan, Italy; 5 Infectious Diseases Section, Louis Stokes Cleveland Department of VA Medical Center, Cleveland, Ohio, United States of America; 6 Department of Experimental Medicine, University of L'Aquila, L'Aquila, Italy; 7 Department of Gastrointestinal Surgery, Hospital Clinic, IDIBAPS, CIBERehd, Barcelona, Spain; 8 Department of Clinical Microbiology, Hospital Clinic, IDIBAPS, CIBERehd, Barcelona, Spain; 9 Research Center for Alcoholic Liver and Pancreatic Diseases, Keck School of Medicine, University of Southern California, Los Angeles, California, United States of America; 10 Department of Gastroenterology and Hepatology, Cleveland Clinic Foundation, Cleveland, Ohio, United States of America; Emory University, United States of America

## Abstract

**Background:**

Probiotics appear to be beneficial in inflammatory bowel disease, but their mechanism of action is incompletely understood. We investigated whether probiotic-derived sphingomyelinase mediates this beneficial effect.

**Methodology/Principal Findings:**

Neutral sphingomyelinase (NSMase) activity was measured in sonicates of the probiotic *L. brevis (LB)* and *S. thermophilus (ST)* and the non-probiotic *E. coli (EC)* and *E. faecalis (EF)*. Lamina propria mononuclear cells (LPMC) were obtained from patients with Crohn's disease (CD) and Ulcerative Colitis (UC), and peripheral blood mononuclear cells (PBMC) from healthy volunteers, analysing LPMC and PBMC apoptosis susceptibility, reactive oxygen species (ROS) generation and JNK activation. In some experiments, sonicates were preincubated with GSH or GW4869, a specific NSMase inhibitor. NSMase activity of *LB* and *ST* was 10-fold that of *EC* and *EF* sonicates. *LB* and *ST* sonicates induced significantly more apoptosis of CD and UC than control LPMC, whereas *EC* and *EF* sonicates failed to induce apoptosis. Pre-stimulation with anti-CD3/CD28 induced a significant and time-dependent increase in *LB*-induced apoptosis of LPMC and PBMC. Exposure to *LB* sonicates resulted in JNK activation and ROS production by LPMC. NSMase activity of *LB* sonicates was completely abrogated by GW4869, causing a dose-dependent reduction of *LB*-induced apoptosis. *LB* and *ST* selectively induced immune cell apoptosis, an effect dependent on the degree of cell activation and mediated by bacterial NSMase.

**Conclusions:**

These results suggest that induction of immune cell apoptosis is a mechanism of action of some probiotics, and that NSMase-mediated ceramide generation contributes to the therapeutic effects of probiotics.

## Introduction

Probiotics are defined as live, non-pathogenic bacteria that confer health benefits beyond their nutritional value [1]. A growing body of evidence, accumulated in the last decade, suggests that probiotics can be useful in the treatment of inflammatory bowel disease (IBD), especially in patients with ulcerative colitis (UC) and pouchitis [2]–[4]. In spite of this clinical evidence, our understanding of the biological processes involved in the beneficial effects of probiotics is still limited. Several mechanisms have been postulated to contribute to the anti-inflammatory effect of probiotics in the gut, including competitive exclusion of pathogens, production of antimicrobial agents and organic acids, enhancement of the epithelial barrier function, increase of mucosal IgA secretion, and modulation of lymphocyte and dendritic cell function [5]–[6]. Abnormalities of T cell function are recognized to play a central role in IBD pathogenesis [7]. Among them, resistance of mucosal T-cells to undergo apoptosis is believed to be a critical event by altering the equilibrium between cell death and proliferation resulting in an excessive accumulation of T-cells in the gut [8]–[11]. The pathogenic importance of defective T-cell apoptosis is supported by the observation that various drugs effective in the treatment of CD promote immune cell apoptosis [12]–[16]. This has led to the notion that induction of apoptosis in the mucosal immune compartment is a key step for successful treatment of IBD [17]. Apoptosis is induced by multiple factors and mediated by various mechanisms [18]. One of them involves sphingomyelinases, a group of enzymes responsible for the conversion of sphingomyelin into ceramide, a powerful second messenger which mediates all forms of apoptosis, including receptor-mediated, stress-induced, and cell detachment [19]. Three main families of sphingomyelinases have been described, acidic (ASMase), alkaline and neutral (NSMase), according to their optimal pH [20], [21]. In relation to probiotics, recent observations described that VSL#3, a probiotic that contains a mixture of eight different strains of bacteria, stimulated mucosal alkaline sphingomyelinase activity, although the molecular downstream targets contributing to the therapeutic effect of VSL#3 were not elucidated [22]. NSMAse is also found in bacteria, yeast and mammalian cells, and is a membrane-bound in mammalian cells but a soluble protein in bacterial cells, with great variations in NSMAse concentrations among different bacterial strains [23]. Since the role of NSMase in the therapeutic benefits of probiotics has not been previously reported and because a large number of bacteria produce NSMase [24] and probiotics can induce apoptosis [25], we hypothesised that the probiotic strains used in the management of IBD might exert an anti-inflammatory effect by inducing immune cell death. If present in adequate concentrations, probiotic NSMase could induce ceramide formation and trigger signalling pathways leading to apoptosis of lamina propria mononuclear cells (LPMC) in patients with IBD, counterbalancing the defective immune cell death. Therefore, the specific aim of this study was to investigate the capacity of probiotics to produce NSMase, and induce ceramide-mediated immune cell apoptosis. Our data indicate that bacterial NSMase-mediated ceramide generation induced immune cell apoptosis via JNK activation and ROS overgeneration, two known targets of ceramide action.

## Results

### Increased NSMase activity in probiotic bacteria

**Figure 1:**
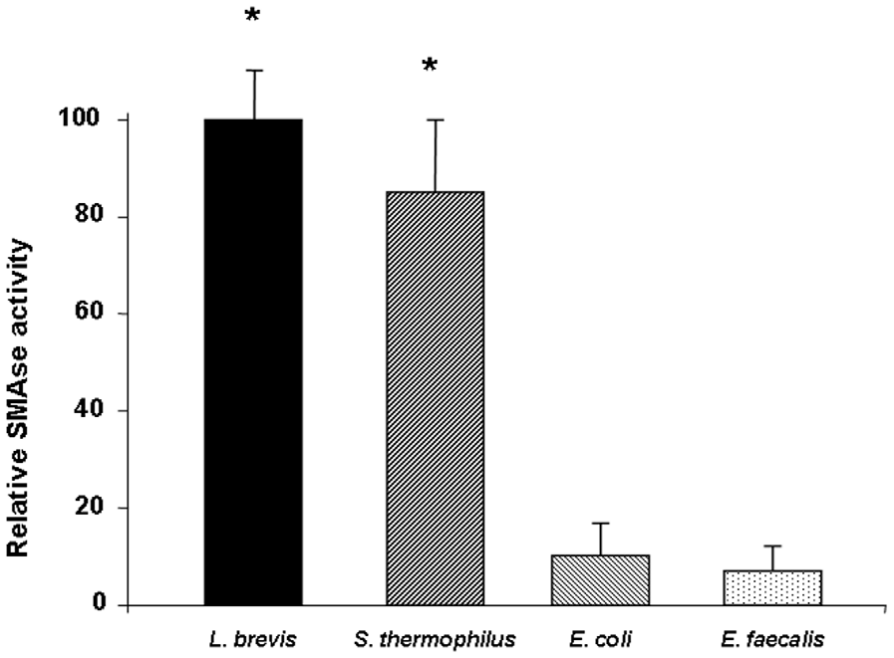
Neutral SMAse activity of bacterial sonicates. Neutral SMAse activity was quantified in *L. brevis*, *S. thermophilus*, *E. coli* and *E. faecalis* sonicates. SMAse activity of each bacterial sonicate is expressed as relative SMAse activity, assigning an arbitrary value of 100 to the mean SMAse activity of *L. brevis* sonicates. Each bar represents 3 separate experiments. *p<0.01 vs. *E. coli* and *E. faecalis*.

Considering that several strains of bacteria can produce different amounts of NSMase [24], we initially measured the NSMase activity in each one of the probiotic and non-probiotic sonicates used in our study. *S. thermophilus and L Brevis* exhibit well documented probiotic properties and are considered probiotic bacteria. S. thermophilus is one of the components of the probiotic mixture VSL#3, which has been used in the treatment of pouchitis and ulcerative colitis, among other conditions [2], [3]. As measured by HPTLC analysis, the sphingomyelinase activity of the two probiotic bacteria, *L. brevis* and *S. thermophilus*, was approximately ten-fold higher (p<0.01) than that of the two non-probiotic commensal bacteria *E. coli* and *E. faecalis* (**Fig. 1**). Determination of ASMase activities indicated similar levels of activation in sonicates of probiotic and non-probiotic commensal bacteria (data not shown).

### Induction of LPMC apoptosis by probiotic but not commensal enteric bacteria

**Figure 2:**
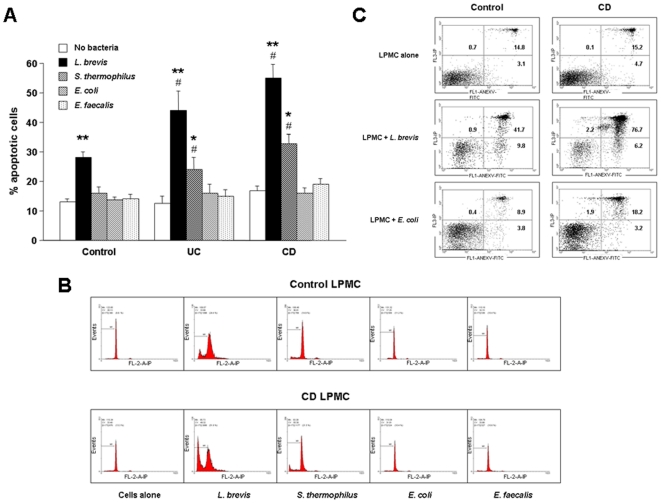
Induction of LPMC apoptosis by bacterial sonicates. A) Control, UC, and CD LPMC were left alone or exposed to *L. brevis*, *S. thermophilus*, *E. coli* or *E. faecalis* sonicates for 24 hours, after which the percentage of apoptotic cells was assessed by PI staining and flow cytometric analysis. Each bar represents 23 control, 7 UC, and 10 CD separate LPMC isolates. * p<0.01 and ** p<0.001 vs. no bacteria; # p<0.05 vs. control LPMC. B) Flow cytometric analysis of control (upper panel) and CD (lower panel) PI-stained LPMC. Figure is representative of 23 control, and 10 CD separate LPMC isolates. C) Flow cytometric analysis of control (left panels) and CD (right panels) Annexin V and PI-stained LPMC. Figure is representative of 4 control, and 4 CD separate LPMC isolates.

In view of the markedly higher levels of NSMAse activity generated by the two probiotic compared to the two non-probiotic bacterial sonicates, we next tested the ability of each bacterium to induce apoptosis of mucosal immune cells. In the absence of bacteria, LPMC exhibited a low rate of spontaneous apoptosis, with no differences among control, UC and CD cells (**Fig. 2a, b**). Exposure of LPMC to the probiotic *L. brevis* sonicate resulted in an increased rate of apoptosis (p<0.001) both in control and IBD cells, the increase being significantly (p<0.05) greater for UC and CD compared to control LPMC (**Fig. 2a**). Similarly, *S. thermophilus* also resulted in an increased LPMC apoptosis (p<0.01), however, this increase was significant (p<0.05) only in UC and CD, but not control cultures (**Fig. 2a**). When the same control, UC and CD cells were exposed to the non-probiotic, non-pathogenic gut commensal *E. coli* or *E. faecalis* sonicates, no significant effect on LPMC apoptosis was found (**Fig. 2a, b**). The pro-apoptotic effect of *L. brevis* and *S. thermophilus* was confirmed in an additional set of experiments in which apoptosis was assessed by double staining with annexin V and propidium iodide (**Fig. 2c**).

### Influence of the cell activation on probiotic-induced immune cell apoptosis

**Figure 3:**
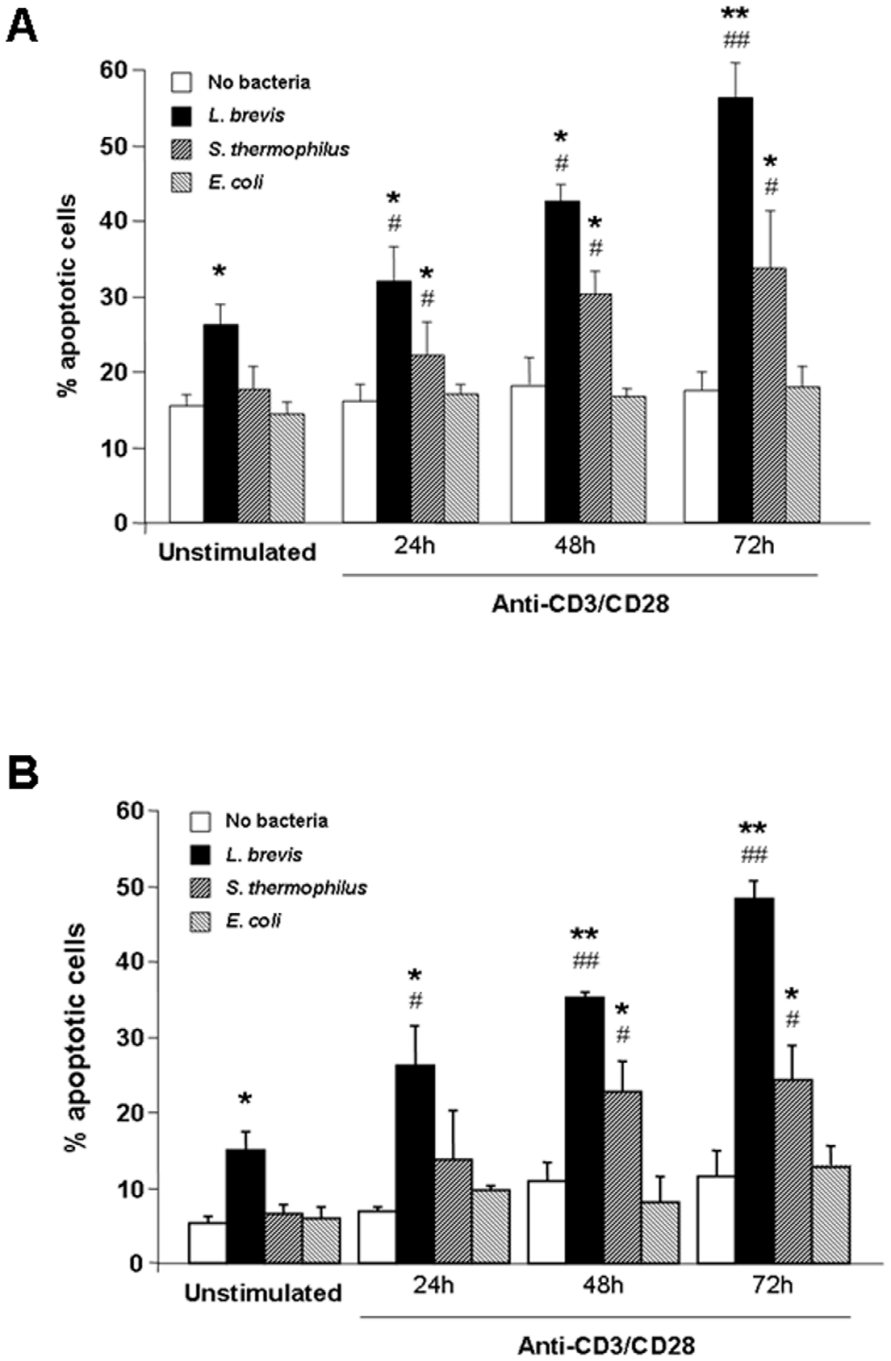
Effect of cell activation on probiotic-induced mononuclear cell apoptosis. A) Control LPMC were left untreated or activated with combined anti-CD3/CD28 for 24, 48 or 72 hours. Subsequently, cells were exposed to *L. brevis*, *S. thermophilus*, or *E. coli* sonicates for 24 hours, after which the percentage of apoptotic cells was assessed. Each bar represents 4 separate experiments. * p<0.01 and ** p<0.001 vs. no bacteria; # p<0.05 and ## p<0.01 vs. unstimulated LPMC. B) Control PBMC were left untreated or activated with combined anti-CD3/CD28 for 24, 48 or 72 hours. Subsequently, cells were exposed to *L. brevis*, *S. thermophilus*, or *E. coli* sonicates for 24 hours, after which the percentage of apoptotic cells was assessed. Each bar represents 4 separate experiments. * p<0.01 and ** p<0.001 vs. no bacteria; # p<0.05 and ## p<0.01 vs. unstimulated PBMC.

The observation that the magnitude of probiotic-induced apoptosis was significantly greater for UC and CD than control LPMC suggests that mucosal cell activation, as found in IBD, may be involved in the process of probiotic-induced apoptosis. To test this hypothesis, we activated freshly isolated control LPMC with anti-CD3/CD28 antibodies for increasing periods of time prior to their exposure to bacterial sonicates. Engagement of the CD3/CD28 pathways resulted in a marked, time-dependent, and significant enhancement of both *L. brevis* and *S. thermophilus*-induced LPMC apoptosis compared to cultures with no bacteria (p<0.01–0.001) and unstimulated LPMC (p<0.05–0.01) (**Fig. 3a**). Interestingly, *E. coli* was unable to upregulate apoptosis of activated LPMC (**Fig. 3a**). To ascertain whether the ability of undergoing probiotic-induced, activation-dependent apoptosis was a distinctive property of LPMC, freshly isolated PBMC were used as an alternate source of immune cells. After activation with anti-CD3/CD28, the pattern of PBMC pro-apoptotic response to both *L. brevis* and *S. thermophilus* resembled that observed with LPMC (p<0.05–0.01) (**Fig. 3b**), confirming that cell activation has a definitive role in probiotic-induced immune cell apoptosis.

### Induction of apoptosis by exogenous ceramide and NSMAse

**Figure 4:**
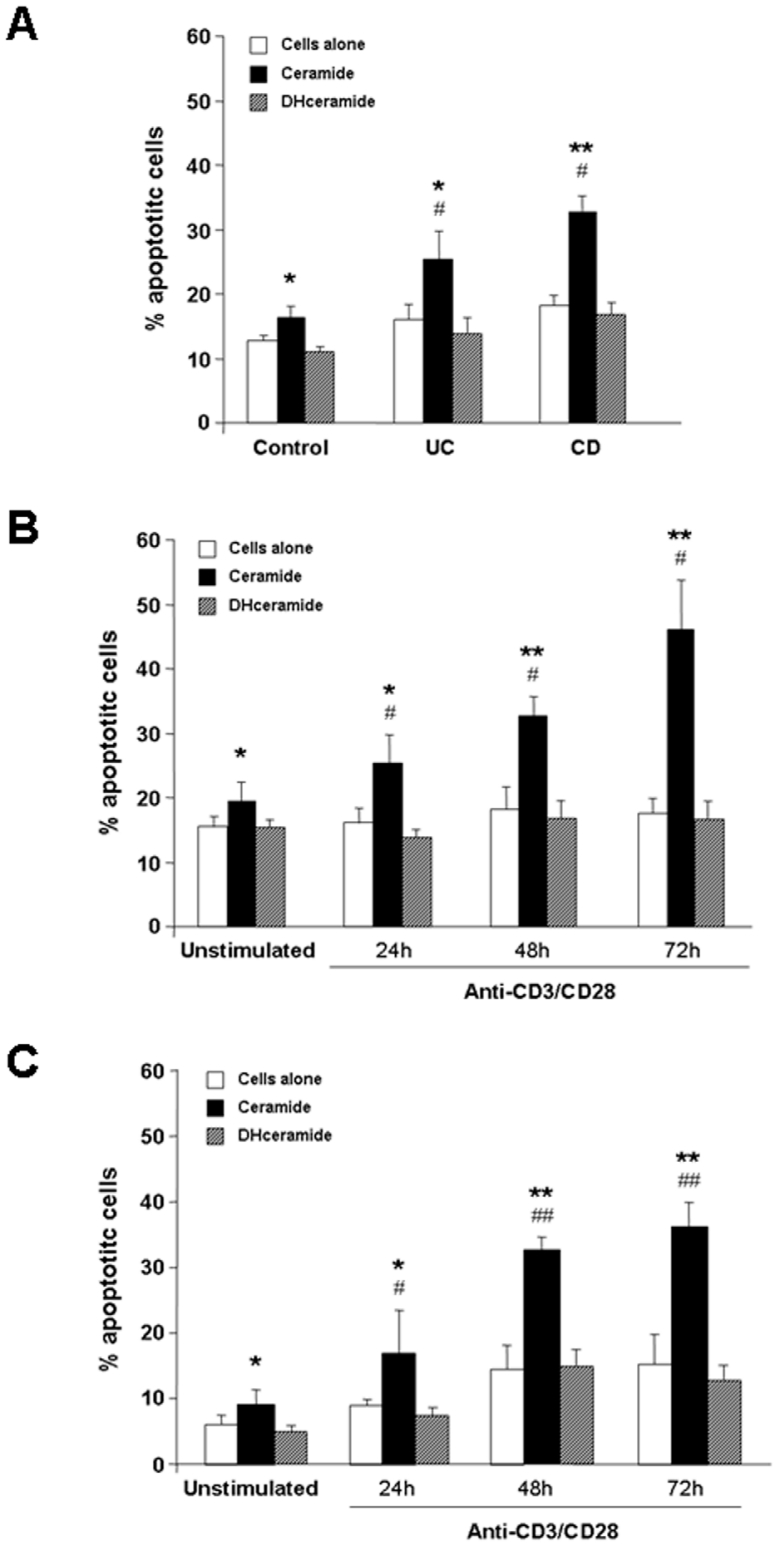
Induction of mononuclear cell apoptosis by exogenous ceramide. A) Control, UC, and CD LPMC were left untreated or cultured with ceramide or dihydroceramide for 24 hours, after which the percentage of apoptotic cells was assessed. Each bar represents 23 control, 7 UC, or 10 CD separate LPMC isolates. * p<0.01 and ** p<0.001 vs. no bacteria; # p<0.05 vs. control LPMC. B) Control LPMC were left untreated or activated with combined anti-CD3/CD28 for 24, 48 or 72 hours. Subsequently, cells were cultured with ceramide or dihydroceramide for 24 hours, after which the percentage of apoptotic cells was assessed. Each bar represents 4 separate experiments. * p<0.05 and ** p<0.01 vs. no bacteria; # p<0.05 vs. unstimulated LPMC. C) Control PBMC were left untreated or activated with combined anti-CD3/CD28 for 24, 48 or 72 hours. Subsequently, cells were cultured with ceramide or dihydroceramide for 24 hours, after which the percentage of apoptotic cells was assessed. Each bar represents 4 separate experiments. * p<0.05 and ** p<0.01 vs. no bacteria; # p<0.05 and ## p<0.01 vs. unstimulated PBMC.

Having detected marked differences in the NSMase activity between probiotic and non-probiotic bacterial sonicates, we next undertook a series of experiments aimed at ascertaining whether the addition of exogenous ceramide to immune cells could reproduce the pro-apoptotic effect observed with probiotic bacteria. Exposure of control, UC and CD LPMC to a pre-determined optimal ceramide concentration (30 µg/mL) resulted in a significant increase of the number of apoptotic cells in all groups (p<0.01–0.001) (**Fig. 4a**). Of note, the degree of apoptosis was significantly greater (p<0.05) in UC and CD than control LPMC (**Fig. 4a**). Dihydroceramide, the inactive precursor of ceramide, failed to induce apoptosis in LPMC regardless of their source either from control, UC or CD patients (**Fig. 4a**). A similar effect was observed when LPMC were exposed to a pre-determined optimal concentration of *Bacillus cereus*-derived NSMAse (data not shown). The pro-apoptotic effect of NSMase required Mg^2+^ supplementation. Ceramide also induced apoptosis in activated LPMC and PBMC (p<0.05) compared to unstimulated cells (see below). Thus, these findings underscore that exogenous ceramide and NSMase reproduce the effects of probiotic sonicates in activated mucosal cells.

### Effect of the cell activation state on ceramide-induced apoptosis

As demonstrated above, cell activation enhanced the degree of probiotic-induced apoptosis. To further corroborate that this effect was mediated by the NSMse/ceramide pathway, we evaluated the effect of cell activation on ceramide-mediated apoptosis following incubation with CD3/CD28 antibodies for 24–72 hours. Anti-CD3/CD28 activation resulted in a marked and time-dependent enhancement of ceramide-induced LPMC apoptosis (**Fig. 4b**), mimicking the previously observed increase in apoptosis of anti-CD3/CD28-activated LPMC by *L. brevis* and *S. thermophilus* (p<0.05–0.01). The pro-apoptotic effect of ceramide was also noted with activated PBMC (p<0.05–0.01) (**Fig. 4c**).

### Activation of JNK and production of ROS in probiotic-triggered LPMC apoptosis

**Figure 5:**
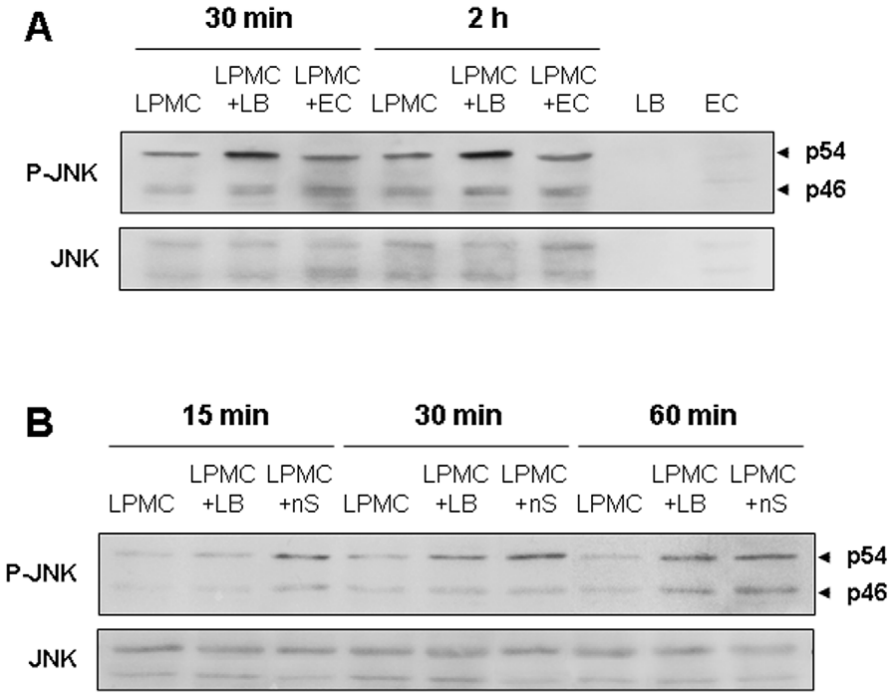
*L. brevis*-induced JNK phosphorylation by LPMC. A) *L. brevis*- but not *E.coli*-induced phosphorylation of JNK in LPMC. Control LPMC were left alone or exposed to *L. brevis* or *E. coli* sonicates for 30 minutes or 2 hours, after which JNK phosphorylation was assessed. JNK is not detected in *L. brevis* or *E. coli* sonicates (two last lanes on the right). B) Exogenous nSMAse-induced phosphorylation of JNK in LPMC. Control LPMC were left alone or exposed to *L. brevis* or recombinant neutral SMAse for 15, 30 or 60 minutes, after which JNK phosphorylation was assessed. LB = *L. brevis* sonicates; EC = *E. coli* sonicates; nS = nSNAse. Figure is representative of 3 separate experiments.

Because ceramide activates stress-activated protein kinases, like c-jun N-terminal kinase (JNK) [26], we investigated whether exposure of control LPMC to *L. brevis* resulted in JNK activation. In a first series of experiments we found that exposure to *L. brevis* sonicates increased JNK phosphorylation in treated compared to untreated LPMC (**Fig. 5a**). In contrast, no increase in JNK phosphorylation was observed when LPMC were exposed to sonicates of the non-probiotic bacteria *E. coli*. JNK was not detected in *L. brevis* or *E. coli* sonicates (**Fig. 5a**). In a second series of experiments, we tested the effect of exogenous NSMase, and we also observed a time-dependent phosphorylation of JNK in LPMC that mimicked the results obtained with *L. brevis* sonicates (**Fig. 5b**). Together, these results further support the involvement of the NSMAse/ceramide pathway in probiotic-induced LPMC apoptosis. Treatment of *L. brevis* sonicates with the specific JNK inhibitor SP-600125 did not prevent the induction of LPMC apoptosis by *L. brevis* sonicates (data no shown), suggesting that other nSMAse-mediated, signalling pathways are able to forward the pro-apoptotic signal.

**Figure 6:**
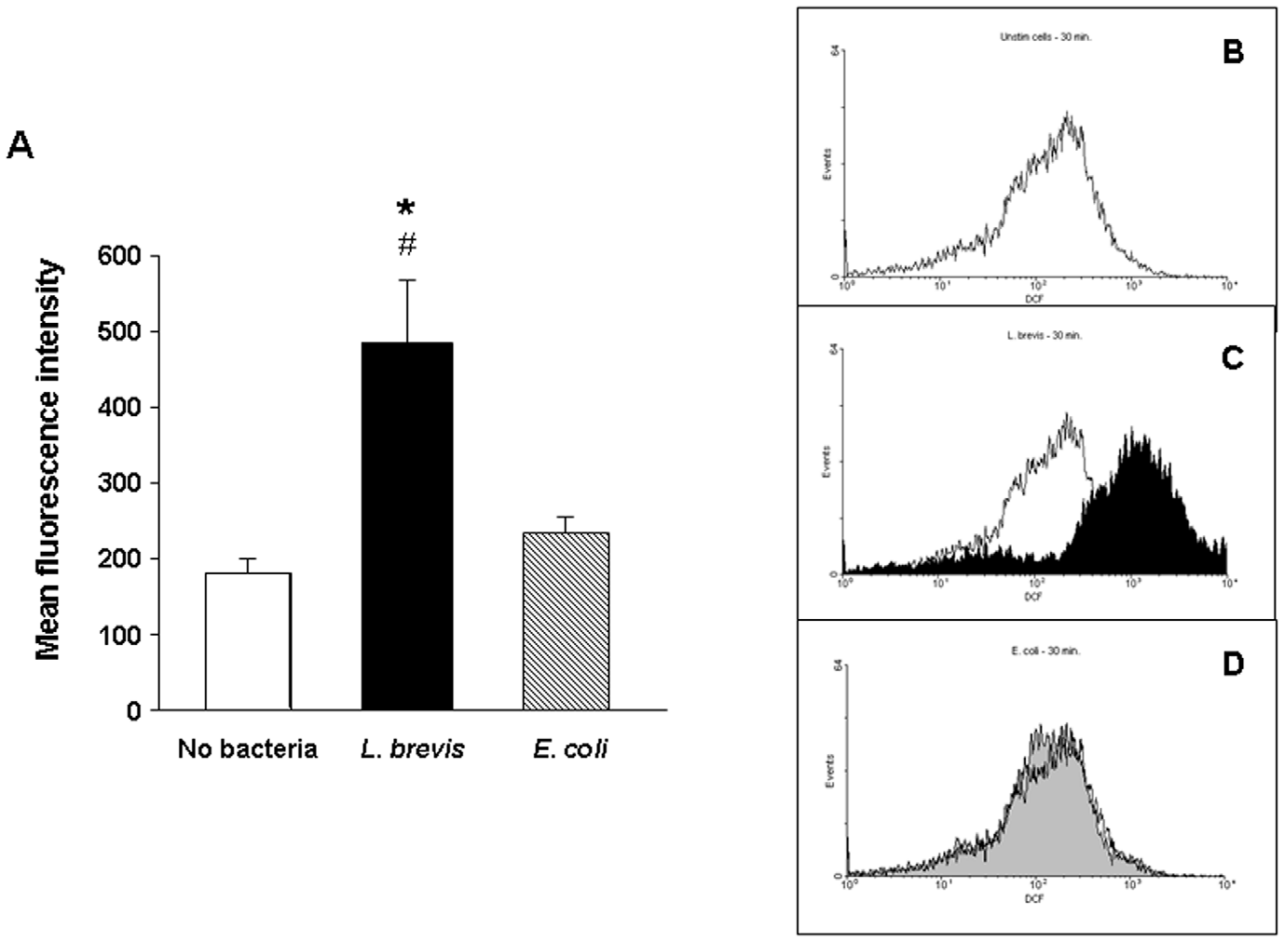
*L. brevis*-induced reactive oxygen species production by LPMC. Left panel (A): control LPMC were left alone or exposed to *L. brevis* or *E. coli* sonicates for 30 minutes, after which production of reactive oxygen species by LPMC was assessed. Data are expressed as mean fluorescence intensity of LPMC. Each bar represents 5 separate experiments. *p = 0.02 vs. no bacteria; #p = 0.04 vs. *E. coli*. Right panel: Flow cytometric analysis of control LPMC. Cells were left alone (B) or exposed to *L. brevis* (C) or *E. coli* (D) sonicates for 30 minutes, after which production of reactive oxygen species by LPMC was quantified and expressed as mean fluorescence intensity. Figures are representative of 5 separate experiments.

Another hallmark of ceramide-mediated cell signalling is the production of reactive oxygen species (ROS) [27], [28], [29]. Exposure of control LPMC to *L. brevis* sonicates resulted in a 3-fold increase (p = 0.02) in ROS production compared to untreated cells (**Fig. 6a**). On the contrary, treatment of the same LPMC with sonicates of the non-probiotic bacteria *E. coli* failed to significantly change LPMC ROS production compared to untreated cells (**Fig. 6a**). Panels b, c and d in Fig. 6 are representative flow cytometry analyses of control LPMC incubated with no bacteria, *L. brevis* or *E. coli* sonicates, respectively. Similar results were obtained when control LPMC were exposed to bacterial sonicates for 60 minutes (data not shown). These results show that ROS are generated during LPMC apoptosis mediated by probiotic nSMAse, further supporting the involvement of the NSMAse/ceramide pathway in this process. Treatment of *L. brevis* sonicates with the phenolic antioxidant butylated hydroxyanisole (BHT), which prevents ROS production, did not influence the induction of LPMC apoptosis by *L. brevis* sonicates (data no shown), suggesting that other NSMAse-mediated, signalling pathways are able to forward the pro-apoptotic signal.

### Blockade of probiotic-induced cell death by inhibition of NSMAse activity

**Figure 7:**
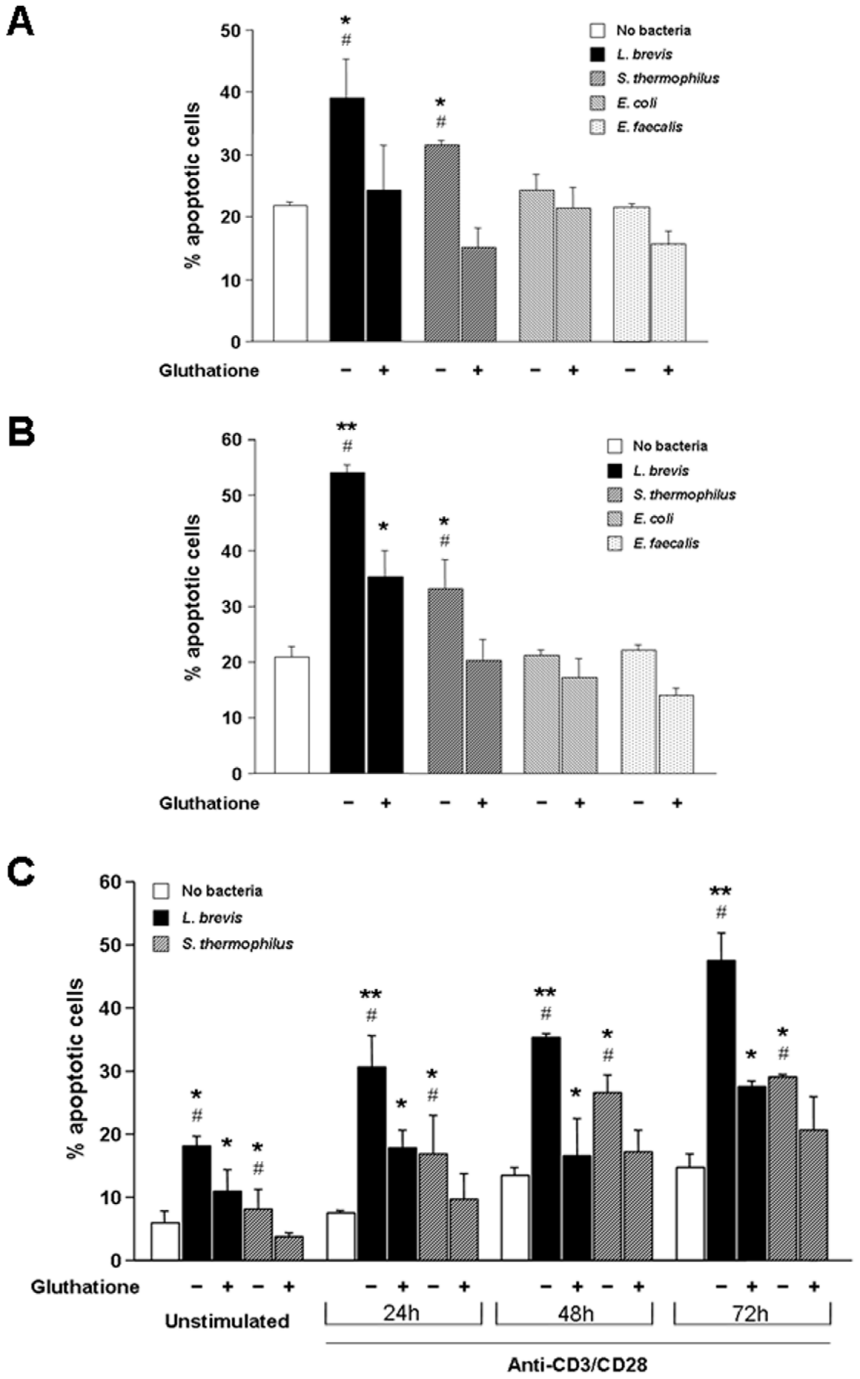
Inhibition of *L. brevis*-induced cell apoptosis by glutathione. A) Control LPMC were left alone, exposed to *L. brevis*, *S. thermophilus*, *E. coli* or *E. faecalis* sonicates, or bacterial sonicates previously treated with 5 mM *glutathione* for 24 hours, after which the percentage of apoptotic cells was assessed. B) CD LPMC were left alone, exposed to *L. brevis*, *S. thermophilus*, *E. coli* or *E. faecalis* sonicates, or bacterial sonicates previously treated with 5 mM *glutathione* for 24 hours, after which the percentage of apoptotic cells was assessed. C) Control PBMC were left unstimulated or activated with combined anti-CD3/CD28 for 24, 48 or 72 hours. Subsequently, cells were exposed to *L. brevis or S. thermophilus* sonicates, or to bacterial sonicates previously treated with 5 mM *glutathione* for 24 hours, after which the percentage of apoptotic cells was assessed. Each bar represents 4 separate experiments. * p<0.05 and ** p<0.01 vs. no bacteria; # p<0.05 vs. no *glutathione*.

**Figure 8:**
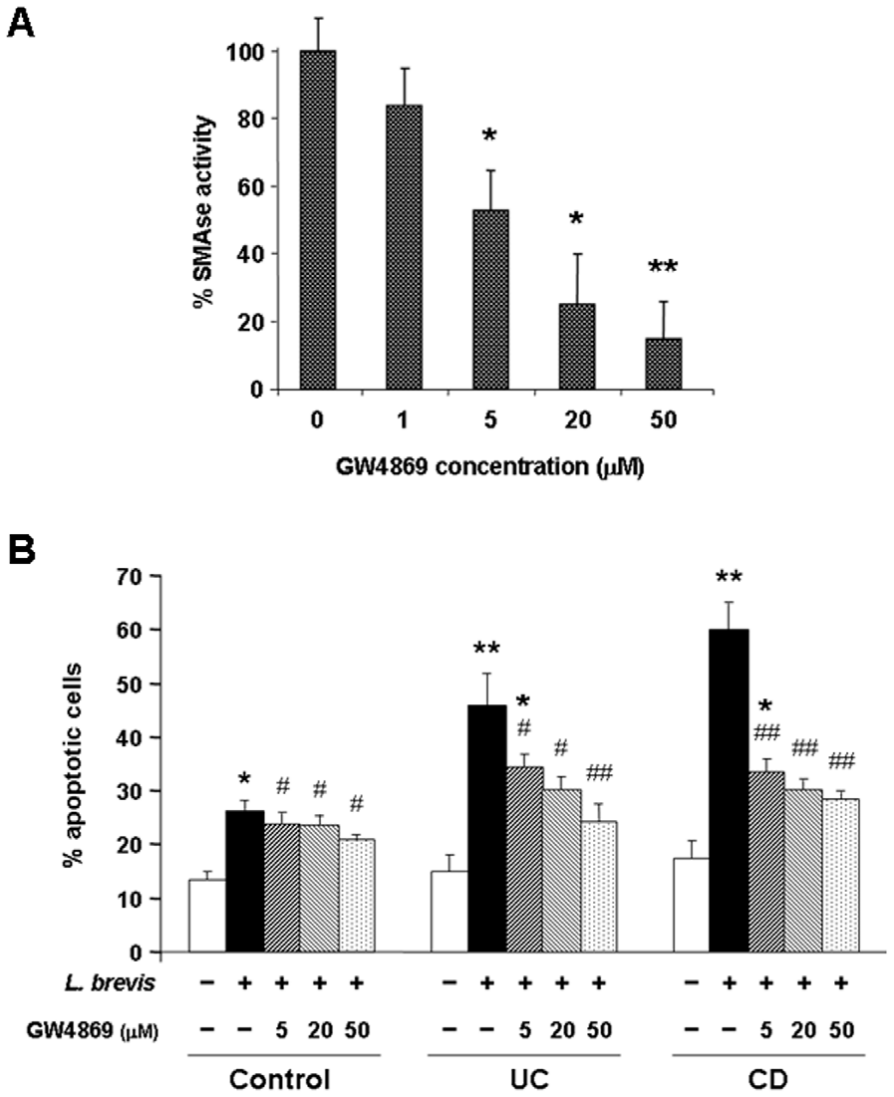
Specific inhibition of *L. brevis*-induced cell apoptosis by GW4869. A) *L. brevis* sonicates were exposed to increasing concentrations of GW4869. SMAse activity of each bacterial sonicate is expressed as relative SMAse activity, assigning an arbitrary value of 100 to the mean SMAse activity of *L. brevis* sonicates. Each bar represents 3 separate experiments. * p<0.05 and ** p<0.01 vs. no GW4869. B) Control LPMC were left alone or exposed to *L. brevis* sonicates previously treated with different doses of GW4869 for 24 hours, after which the percentage of apoptotic cells was assessed. Each bar represents 4 separate LPMC isolates. * p<0.05 and ** p<0.01 vs. no bacteria; # p<0.05 and ## p<0.01 vs. no GW4869.

The results so far point to NSMAse as the mediator responsible for the pro-apoptotic effects of probiotic bacterial sonicates. To confirm this notion, we performed a series of blocking experiments using GSH, which is known to inhibit NSMase [30]. When control LPMC were exposed to sonicates of either *L. brevis* or *S. thermophilus* previously preincubated with GSH, a complete abrogation of the sonicates' pro-apoptotic effect was observed (p<0.05) (**Fig. 7a**). A similar effect was also observed with CD LPMC, with a significant (p<0.05) reduction in the percentage of apoptotic cells with both *L. brevis* and *S. thermophilus* GSH-treated sonicates (**Fig. 7b**). In contrast, preincubation of *E. coli* or *E. faecalis* sonicates with GSH had no significant modulatory effect on control or CD LPMC apoptosis (**Fig. 7a,b**). GSH also inhibited the pro-apoptotic effect of *L. brevis* and *S. thermophilus* on resting and CD3/CD28-activated PBMC (**Fig. 7c**). Although the capacity of GSH to abrogate the activity of NSMase is well established, GSH could have other effects on bacterial sonicates that might affect cell apoptosis. Therefore, we undertook additional experiments using GW4869, a specific inhibitor of NSMase [31]. Preincubation of *L. brevis* sonicates with various concentrations of GW4869 resulted in a clear dose-dependent inhibition of NSMase activity (p<0.05–0.01) (**Fig. 8a**). When an optimal inhibitory dose of GW4869 was added to *L. brevis* sonicates a marked and significant (p<0.05–0.01) reduction of their capacity to induce LPMC apoptosis was observed, more so for UC and CD than control LPMC (**Fig. 8b**). The combined results of the blocking experiments confirm the essential role of the NSMAse/ceramide pathway in mediation of probiotic-induced LPMC apoptosis.

## Discussion

The molecular mechanisms of the beneficial effects of probiotics remain to be fully elucidated. Recent observations showed that VSL#3, a probiotic containing a mixture of bacteria, stimulated mucosal alkaline sphingomyelinase and reduced inflammation [22]. However, the underlying mechanisms and downstream targets for the putative role of alkaline sphingomyelinase in mediating the therapeutic effects of probiotics were not characterized [22]. The present study shows that *L. brevis* and *S. thermophilus*, two probiotic bacterial sonicates, can induce apoptosis of mucosal immune cells through the SMAse/ceramide pathway. This effect is not shared by *E. coli* and *E. faecalis*, the two non-probiotic enteric bacterial sonicates studied, suggesting that the ability to kill immunocytes is restricted to bacterial strains containing sufficient quantities of the key enzyme NSMase. We show that extracts of the probiotic *L. brevis* or *S. thermophilus* induced apoptosis of systemic and mucosal immune cells. Not only was this effect greater in magnitude than that of the non-probiotic *E. coli* or *E. faecalis*, but was also greater for LPMC from IBD than control non-IBD mucosa. Because IBD cells are in an enhanced state of activation, activation or differentiation may be a requirement to render immune cells susceptible to the pro-apoptotic effect of probiotics. This appears to be the case, as CD3/CD28-stimulated LPMC and PBMC displayed increased apoptosis upon exposure to *L. brevis* or *S. thermophilus* sonicates. The degree of cell activation, as determined by the duration of CD3/CD28 stimulation, correlated closely with the amount of mononuclear cell apoptosis, underscoring the importance of cell activation for probiotic-mediated cell death. These results are in keeping with previous evidence showing a tight relationship between immune cell activation and apoptosis. Activation-induced cell death is a complex physiological event aimed at deleting an excess of activated lymphocytes to prevent autoimmunity or uncontrolled immune reactivity [18], [32]. The relevance of this mechanism to autoimmunity prevention and maintenance of tolerance is exemplified by the development of autoimmune diseases in mice and humans with inherited defects in Fas or FasL [33], [34].

Because probiotics are live bacteria with a vast array of components and functions, the exact mechanisms explaining the beneficial effects are complex and difficult to dissect. If the capacity to induce immune cells apoptosis is an exclusive property of some probiotic bacteria, as found in the present study, certain products with the ability to induce apoptosis should be predominantly expressed by these probiotics. The striking quantitative difference in NSMAse activity between the two probiotic and the two commensal bacterial sonicates included in our study clearly points towards this enzyme as the likely culprit for the pro-apoptotic action of *L. brevis* and *S. thermophilus*. To test this possibility a series of experiments was carried out.

First, *L. brevis* and *S. thermophilus* but not *E.coli* or *E. faecalis* sonicates exhibited enhanced NSMase activity. Further, we demonstrated that exposure of resting and activated PBMC and LPMC, to either exogenous ceramide or recombinant NSMAse results in a marked enhancement of cell apoptosis, mimicking the effect seen with the probiotic strains *L. brevis* and *S. thermophilus*. As negative control, the inactive sphyngolipid dihydroceramide, failed to induce apoptosis. Corroborating evidence was obtained by showing that the level of ceramide-induced apoptosis was also dependent of the degree of immune cell activation.

Second, we investigated some of the ceramide-triggered pathways during probiotic-induced mononuclear cell apoptosis. Signalling downstream of ceramide is extremely complex, but two of the best-known pathways involve JNK activation [26], [35] and ROS production [27], [36]. As we hypothesised, phosphorylation of JNK was observed after exposure of LPMC to *L. brevis*, but not to the commensal *E. faecalis*. Of note, exposure of LPMC to recombinant NSMAse also induced JNK activation, and to a similar degree than *L. brevis* sonicates. Likewise, *L. brevis*, but not *E. faecalis*, induced a four-fold increase in ROS production by LPMC. The selectivity of *L. brevis* effects on JNK activation and ROS formation bolster the notion that NSMAse, present in large quantities in probiotics, and its product ceramide are intimately involved in the process of probiotic-induced immune cell apoptosis. The fact that neither JNK nor ROS production blockade separately were able to prevent probiotic sonicate-induced LPMC apoptosis reflects the complexity of signalling downstream of ceramide, suggesting the existence of redundant pathways to forward the pro-apoptotic signal. For instance, in addition to JNK/ROS, ceramide (independently of its mechanism of generation) can target other cell death regulators, such as cathepsin D or protein phosphatase PP1 or PP2, among others [19].

Third, we investigated the consequences of NSMAse inhibition, which should result in a reduction of the probiotic pro-apoptotic capacity. Preincubation of *L. brevis* and *S. thermophilus* sonicates with optimal concentrations of GSH, a potent inhibitor of NSMAse activity [30], markedly reduced apoptosis. A potential limitation to the interpretation of these results is that GSH, besides nSMAse inhibition, could influence the ability of probiotic sonicates to induce apoptosis by modifying the redox state [37]. To address this issue, we used the highly specific nSMAse inhibitor GW4869, which does not influence the redox state [31]. GW4869 almost completely abrogated in a dose-dependent fashion the nSMAse activity of *L.brevis*, the strain with the highest SMAse activity. Additionally, pre-incubation of *L. brevis* sonicates with optimal doses of GW4869 obviously reduced *L. brevis*-induced LPMC apoptosis, an inhibitory effect more evident for IBD than control LPMC.

Our results cannot be extrapolated to all probiotic agents described to date, since only two of them, *L. brevis* and *S. thermophilus*, were evaluated in our study. The fact that other probiotic microorganisms might have low or absent NSMAse activity, a key molecule for probiotic-induced immune cell apoptosis, as shown in our study, implies that the assessment of NSMAse activity may have clinical applications, helping the investigators to select the best probiotics to be tested in IBD.

The relevance of our findings mostly rests on the concept that resistance of mucosal immune cells to apoptosis is considered an essential component of IBD pathogenesis (17), probably in combination with an excessive cell proliferation [38], [39]. The clinical relevance of immune cell apoptosis in IBD is supported by evidence showing that induction of apoptosis is a mechanism common to most IBD therapies, including aminosalycilates (12), steroids (13), immunosupressants [40], and infliximab [41]. In conclusion, the ability of *L. brevis* and *S. thermophilus* to promote apoptosis may compensate the deficient cell death and thus contribute to re-establish mucosal immune homeostasis and decrease inflammation in IBD-involved intestine.

## Materials and Methods

### Bacterial strains and preparation of bacterial sonicates

Bacterial strains used in this study included the probiotic bacteria *L. brevis* (CD2; DSM#11988; Deutsche Sammlung von Mikroornanisgmen und Zellkulturen, Braunschweig, Germany) and *S. termophilus* (CD8; DSM#14667), and the non-probiotic non-pathogenic bacteria *E. coli* (ATCC#49420; ATCC, Manassas, VA) and *E. faecalis* (ATCC#700802; ATCC). Bacterial suspensions were prepared from cultures of each individual strain. *L. brevis* and *S. thermophilus* were grown in De Man Rogosa Sharp medium (MRS; Difco, Detroit, MI). *E.coli* and *E. faecalis* were grown in a 1% tryptone peptone medium (Difco), 0.5% NaCl, and 0.5% yeast extract (Difco). Bacteria were harvested at the end of logarithmic phase by centrifugation, suspended in four times their wet weight in desintegration buffer containing (7.8 g/l NaH_2_PO_4_, 7.1 g/l Na_2_HPO_4_, 0.247 g/l MgSO_4_×7H_2_O, 1 mM EDTA, 1 mM DTT, proteases inhibitors, and triton) and, after three freeze-thaw cycles, sonicated (30 min, alternating 10 s of sonication and 10 s of pause) with a Vibracell sonicator (Sonic and Materials, Danbury, CT).

### Isolation of human LPMC and PBMC

Lamina propria mononuclear cells (LPMC) were isolated from surgical specimens obtained from patients admitted for bowel resection for UC and CD, while malignant and nonmalignant conditions provided histologicaly normal control tissue. All diagnoses were confirmed by clinical, radiologic, endoscopic, and histologic criteria. LPMC were isolated as previously described [42]. Blood samples were collected from patients with UC and CD, as well as healthy subjects. Peripheral blood mononuclear cells (PBMC) were isolated from heparinized venous blood, using a Ficoll-Hypaque density gradient, as previously reported [43]. This project was approved by the Institutional Review Board of University Hospitals of Cleveland and Hospital Clinic i Provincial of Barcelona.

### Flow-cytometric characterization of mononuclear cell apoptosis

A series of preliminary studies were carried out to ascertain the optimal concentration of bacterial sonicates and time of exposure of mononuclear cells to induce apoptosis. In subsequent experiments, bacterial sonicates were added to mononuclear cell cultures at a concentration of 1 mg protein of bacterial sonicate/10^6^ cells/ml, for 24 h. Apoptosis and cell cycle phase distribution were analyzed by propidium iodide staining followed by flow cytometry, as previously described [38]. Where indicated, LPMC and PBMC were pre-stimulated with CD3 monoclonal antibody (mAb) (OKT3; Ortho Diagnostic System INC., Raritan, NJ) and CD28 mAb (Pharmingen Biosciences, San Diego, CA, USA) for different periods of time (24, 48 or 72 hours) before exposure to the bacterial sonicates. Similarly, where indicated, *L. brevis* sonicates were preincubated for 30 min with 5 mM glutathione (GSH) (Calbiochem) or various concentrations of the specific NSMAse Inhibitor GW4869 (Sigma, St. Louis, MO), before addition to the cell cultures. In an additional set of experiments, apoptosis was assessed by combined annexin V/propidium iodide staining (Dako, Glostrup, Deenmark), according to the manufacturer's instructions. In some cases, LPMC and PBMC were incubated with various concentrations of C2-ceramide (Biomol International, Plymouth Meeting, PA), the inactive sphingolipid dihydroceramide (Biomol International), or nSMase from *Bacillus cereus* (Sigma) and examined for apoptosis as described above.

### Quantification of the NSMase activity of bacterial sonicates

Lyophilized bacteria (10 mg) were resuspended and sonicated as described above. Protein concentration was determined by the microbicinchoninic acid assay kit (Pierce, Rockford, IL) with bovine serum albumin standards. Different amounts of bacterial sonicates (1, 0.5, and 0.25 mg) in 50 mM Tris·HCl buffer, pH 7.4, containing 5 mM MgCl_2_ and [*N*-*methyl*-14C]sphingomyelin (0.28 µCi/ml, specific activity 55 mCi/mmol; Amersham, Buckinghamshire, UK), were used for determination of NSMase activity as described before [44]. Alternatively, NSMase activity was also determined by incubating extracts with C6-7-nitro-2,1,3-benzoxadiazol-4-yl (NBD)-sphingomyelin analyzing NBD-ceramide by TLC [45]. Determination of ceramide levels was performed by the diacylglycerol (DAG) kinase assay as described [27]. In some instances, ceramide levels were analyzed by HPLC after deriatization of the sphingoid base with O-phthaldehyde following deacylation of ceramide as described previously [46]. Where indicated, the bacterial preparations were preincubated for 30 min with 5 mM GSH [30] or various concentrations of GW4869 to inhibit nSMase activity, as previously described [31].

### JNK activation in LPMC

At the end of 24 hr culture with bacterial sonicates, cells were washed, lysed and 40 µg of protein were run on a 10% SDS-PAGE and transferred onto a nitrocellulose membrane at 140 mA in 25 mM Tris-HCl, pH 8.3, 192 mM glycine, and 20% methanol. To study the activation of JNK, western blot analysis was performed using an anti-phosphorylated JNK antibody, dilution 1∶1000 (Pharmingen Biosciences, San Diego, CA), according to the manufacturer's instructions. In additional experiments, we also assessed the functional impact of JNK blockade on probiotic sonicates-induced LPMC apoptosis. The specific JNK inhibitor SP-600125 was purchased from Calbiochem (San Diego, CA, USA) and dissolved in dimethyl sulfoxide (DMSO, Sigma-Aldrich). Stock solutions of at least 20 mM were made. *L. brevis* sonicates were incubated with SP-600125 for 30 minutes, before its addition to LPMC cultures. LPMC apoptosis and cell cycle phase distribution were analyzed by PI staining followed by flow cytometry, as described above.

### Reactive oxygen species (ROS) production in LPMC

Hydrogen peroxide was measured by cytometry using chloromethyl-2′-7′-dichlorofluorescein diacetate (DCF; Invitrogen Molecular Probes, Eugene, OR). LPMC were incubated with the fluorescent probe at 2 mM, in the absence or presence of bacterial sonicates. Fluorescence was determined at 529 nm for emission and 503 nm for excitation, with slit widths of 10 and 5 nm, respectively, after 30 and 60 minutes of LPMC exposure to bacterial sonicates [47], [48]. Fluorescence of DCF was correlated with increasing concentrations of hydrogen peroxide allowing determination of hydrogen peroxide, as described [47]. In additional experiments, we also assessed the functional impact of ROS production blockade on probiotic sonicates-induced LPMC apoptosis. To prevent ROS production the phenolic antioxidant butylated hydroxyanisole (BHT) was obtained from Sigma (St. Louis, MO, USA) and added to bacterial sonicates for 30 minutes, before its addition to LPMC cultures. LPMC apoptosis and cell cycle phase distribution were analyzed by PI staining followed by flow cytometry, as described above.

### Statistical Analysis

Data were analyzed by Stat View software (SAS Institute Inc., Cary, NC) using the non-parametric Kruskall-Wallis and Mann-Whitney tests. Repeated measures for the same subject were analyzed by using the Student paired *t* test. Values are expressed as the mean ± SEM. Statistical significance was set at a P value of less than 0.05.
