# Correction: Probiotic Sonicates Selectively Induce Mucosal Immune Cells Apoptosis through Ceramide Generation via Neutral Sphingomyelinase

**DOI:** 10.1371/journal.pone.0278452

**Published:** 2022-11-28

**Authors:** The PLOS ONE Editors

The Competing Interests statement for this article [1] is incomplete. Based on information gathered in editorial follow up, an updated Competing Interests statement is as follows: At the time of publication of this study, the probiotic formulation sold as VSL#3 was produced by Dupont/Danisco in the US. Claudio De Simone (CDS) is the inventor of this probiotic formulation (also known as the De Simone Formulation). CDS is named as an inventor and assignee on patent “Dietary and pharmaceutical compositions containing lyophilized lactic bacteria, their preparation and use” (U.S. Patent No. 5,716,615) and the sole owner of the knowhow used to formulate the product. At the time of publication of the article, CDS owned one share of stock in, and served as Chief Executive Officer and Director for, VSL Pharmaceuticals, Inc., which sold the De Simone Formulation until 2016. MGC was previously a paid consultant for VSL Pharmaceuticals. This study does not use products provided by VSL Pharmaceuticals or Danisco. The strain of *S*. *thermophilus* used in this study is different to the strain included in the De Simone Formulation of VSL#3. This does not alter the authors’ adherence to *PLOS ONE* policies on sharing data and materials.

MGC and CDS disagree that the above information falls within the scope of the interests that must be declared for this article per the journal’s editorial policy.
